# Morphological and biochemical characterization of bacterial isolates from diverse soil habitats in the Amhara region, Ethiopia

**DOI:** 10.1515/biol-2025-1379

**Published:** 2026-09-25

**Authors:** Tirusew Sema Kebede, Destaw Damtie, Mulugeta Kibert, Fasika Melese, Amare Bitew, Mastewal Alehegn

**Affiliations:** Department of Biology, College of Science, Bahir Dar University, P.O. Box 79, Bahir Dar, Ethiopia; Department of Biology, College of Science/Sine Hosaena Center, Bahir Dar University, P.O. Box 79, Bahir Dar, Ethiopia

**Keywords:** soil bacteria, biochemical characterization, bacterial isolates, Shannon diversity index, serial dilution

## Abstract

Soil harbors diverse microbial communities that play essential roles in ecosystem functioning; however, many microbial populations remain unexplored. In this study, bacterial isolates obtained from 11 selected soil sampling sites in the Amhara region of Ethiopia were characterized. A total of 104 morphologically distinct bacterial isolates were recovered using the serial dilution and spread plate techniques and were subsequently characterized based on their morphological and biochemical characteristics. Presumptive identification based on morphological and biochemical characteristics classified the isolates into nine bacterial genera. The Shannon diversity index (H′ = 1.68) indicated moderate bacterial species diversity, whereas the Simpson diversity index (0.72) suggested a relatively even distribution of taxa within the sampled soils. The present study demonstrated that soil bacterial diversity was significantly and positively correlated with soil pH, total nitrogen (TN), and available phosphorus (AP). In contrast, organic carbon (OC), soil moisture, and temperature showed no significant association with bacterial diversity. Principal component analysis (PCA) explained 73 % of the total variation in the biochemical characteristics of the bacterial isolates. The high enzymatic activity and metabolic versatility exhibited by the isolates highlight their potential for various biotechnological applications, including bioremediation, industrial enzyme production, and agricultural biofertilizer development.

## 1 Introduction

Soil is one of the most diverse terrestrial habitats, harboring an immense diversity of microorganisms that play essential roles in maintaining ecosystem functions [1]. Soil microorganisms are key drivers of nutrient cycling, organic matter decomposition, humus formation, and overall soil fertility, making them indispensable to ecosystem functioning [2]. The major groups of soil microorganisms include bacteria, fungi, archaea, actinomycetes, and algae, among which bacteria are the most abundant and metabolically diverse [3]. The abundance, diversity, and composition of soil microbial communities are influenced by both biotic factors, such as vegetation type, plant diversity, and microbial interactions, and abiotic factors, including soil structure, nutrient availability, pH, moisture content, climate, and temperature [4]. These environmental factors shape microbial community composition by selecting for bacteria with distinct morphological, physiological, and biochemical characteristics, which can be used for preliminary identification and classification [5].

Morphological characterization involves the examination of colony characteristics, Gram reaction, cell shape, motility, and endospore formation, providing a rapid approach for the preliminary identification of bacterial isolates. Biochemical characterization includes tests such as catalase activity, methyl red, citrate utilization, indole production, triple sugar iron (TSI) agar, starch hydrolysis, urease activity, and hydrogen sulfide (H_2_S) production. These biochemical tests provide insights into bacterial metabolic capabilities and aid in differentiating among various bacterial genera. Although these techniques are considered classical microbiological approaches, they remain indispensable first-line tools for assessing microbial diversity, particularly in understudied regions where access to molecular identification facilities is limited [6], 7]. Furthermore, these methods are rapid, simple, and cost-effective for preliminary identification and facilitate the selection of promising isolates for subsequent molecular characterization and downstream applications [7].

Each environment presents unique selective pressures that shape resident bacterial communities and influence their ecological functions and observable characteristics. The composition, abundance, and diversity of soil bacterial communities differ between forest interiors and forest edges due to variations in microclimatic conditions and soil properties [8]. Owing to increased environmental stress, enhanced microbial turnover, and differences in nutrient availability, forest edge soils may support more metabolically active bacterial communities, including antibiotic-producing taxa, compared with forest interior soils [8]. Rice field soils harbor diverse bacterial communities, particularly members of the phyla *Actinobacteria* and *Proteobacteria*, with *Bacillus* species frequently reported as dominant taxa occurring as both free-living and plant-associated forms [9]. Solid waste disposal sites are also considered potential reservoirs of bacteria belonging to genera known to include antibiotic-producing species, particularly members of *Actinobacteria* and *Bacillus* [10]. Tannery wastewater and contaminated disposal sites are characterized by elevated concentrations of chromium, high chemical oxygen demand (COD), and increased salinity, creating selective conditions that favor bacteria possessing heavy metal resistance mechanisms, robust cell structures, and specialized metabolic pathways for adaptation and survival [11]. Similarly, animal husbandry waste disposal sites represent important reservoirs of antibiotic residues, creating selective conditions that favor the persistence and dissemination of antibiotic-resistant bacteria as well as the occurrence of antibiotic-producing microorganisms [12]. The high organic matter content and nutrient availability in these environments support the growth of fast-growing, metabolically versatile, and fermentative bacteria, whose physiological characteristics can be assessed through sugar fermentation tests. Freshwater sediments and silt soils harbor diverse bacterial communities, including several genera with reported antimicrobial-producing potential, such as *Actinomycetota* (formerly *Actinobacteria*), *Bacillus*, *Pseudomonas*, *Micromonospora*, *Brevibacillus*, *Paenibacillus*, and *Chitinimonas* [13].

Despite Ethiopia’s recognition as a biodiversity hotspot, the microbial diversity of its diverse ecosystems, ranging from highland forests to agricultural lowlands, remains largely unexplored. In particular, the Amhara region lacks comprehensive baseline information on soil bacterial diversity and community composition, limiting efforts in microbial bioprospecting, ecological assessment, and environmental monitoring. This knowledge gap is increasingly important given the growing anthropogenic pressures associated with agricultural intensification, urban expansion, and industrial development. The region encompasses a wide range of ecosystems that provide valuable opportunities for studying soil microbial diversity, including highland forests such as Tara Gedam, rice cultivation areas in Fogera, and the shoreline environments of Lake Tana, the source of the Blue Nile. These ecosystems, extending from wetlands and agricultural lowlands to rocky mountainous landscapes, including the Simien Mountains (reaching elevations of approximately 4,543 m), and highland plateaus above 1,500 m, harbor diverse microbial communities adapted to varying environmental conditions and ecological stresses, including grazing pressure, agricultural disturbance, and soil erosion.

Therefore, this study aimed to: (1) isolate bacteria from diverse soil habitats in the Amhara region; (2) characterize the morphological and biochemical properties of the bacterial isolates; and (3) identify the bacterial genera and assess the diversity and distribution of culturable bacterial isolates across different sampling sites. This study provides important baseline information on microbial resources and facilitates the selection of promising isolates for further investigation of their antimicrobial potential.

## 2 Materials and methods

### 2.1 Description of the study area

**Figure 1:**
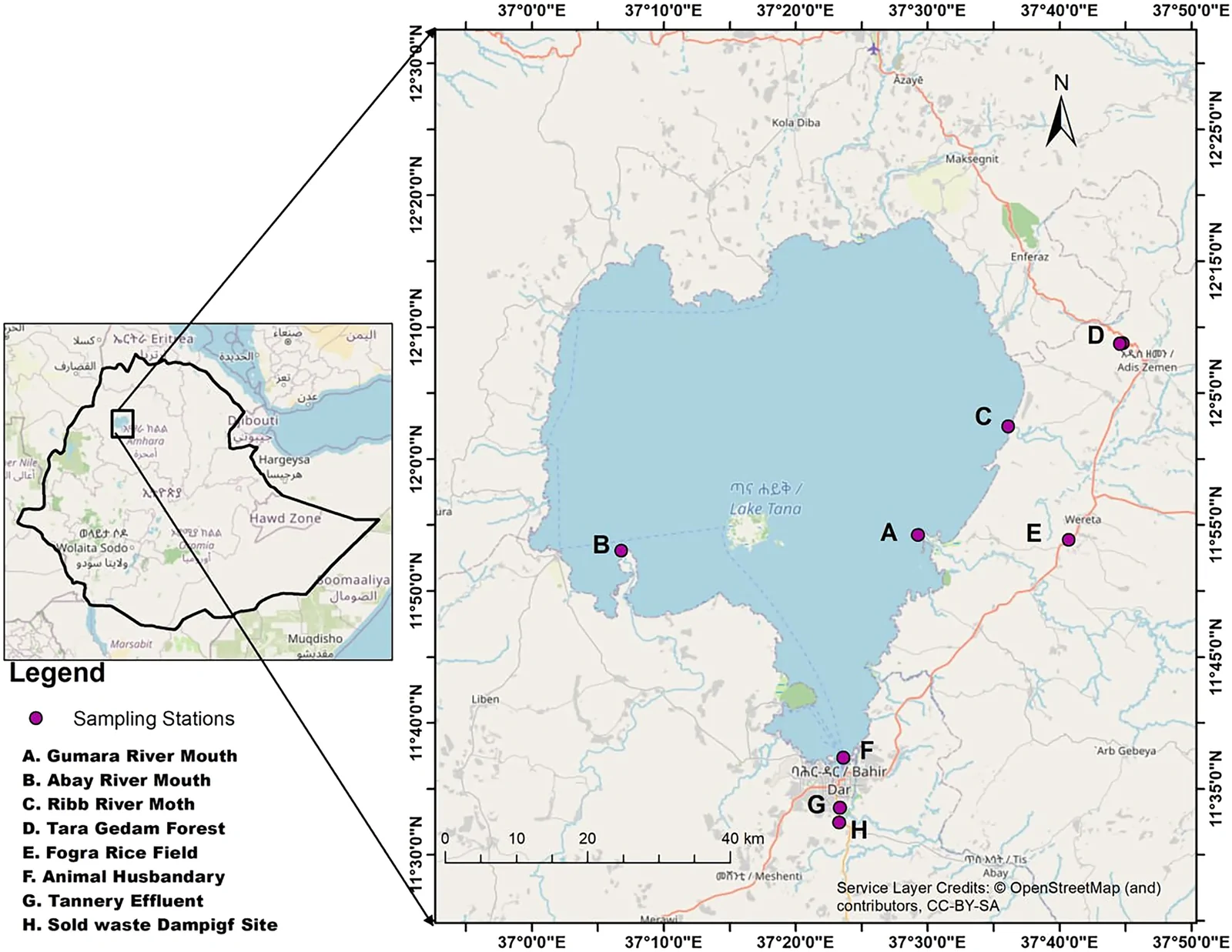
Soil sampling sites across the Amhara region, Ethiopia. Locations: TF = Tara Gedam forest, FI = Fogera inner, FO = Fogera outer, SW = municipal Solid waste, AH = Animal husbandry, TE = Tannery effluent, AB = Abay river mouth, RB = Rib river mouth, GU = Gumara river mouth.

The study was conducted in the Amhara region of northwestern Ethiopia, located between 9° and 14° N latitude and 36° and 40° E longitude (Figure 1). The region is classified into three major agro-climatic zones: highland (>2,300 m above sea level), semi-highland (1,500–2,300 m above sea level), and lowland (<1,500 m above sea level), which account for approximately 20 %, 44 %, and 28 % of the total area of the region, respectively [14].

Soil samples were collected from purposively selected sampling sites representing diverse ecological environments. These sites included Tara Gedam Forest (12°04.351′–12°10.926′ N), located in Libo Kemkem District [15]; the Fogera rice cultivation area (11°53′ N and 37°40′ E); and sediment soils collected from the mouths of rivers draining into Lake Tana, including Rib River (11°41.870′ N and 37°29.564′ E) and Gumara River (11°46.317′ N and 37°29.564′ E) in Fogera District. Additional samples were collected from Abay River in North Achefer District and from selected anthropogenically influenced sites in Bahir Dar City, including a municipal solid waste disposal site (11°32′ N and 37°23′ E), an animal husbandry waste disposal site (11°33′ N and 37°23′ E), and a tannery effluent disposal site (11°37′ N and 37°23′ E).

### 2.2 Collection of soil samples

Soil samples were collected from the study area between March and June 2024. At each of the 11 selected sampling sites, representing contrasting environmental settings, triplicate soil samples were collected at 50 m intervals. The collected samples were then thoroughly mixed to form composite samples, reducing microsite variability and providing a representative sample for each site. A total of 11 composite soil samples were obtained: two from Fogera rice cultivation areas (inside and outside cultivated fields), three from Tara Gedam Forest (forest interior, edge, and outer forest zones), three from anthropogenically influenced sites in Bahir Dar City (animal husbandry waste disposal site, municipal solid waste disposal site, and tannery effluent discharge site), and three from sediment collection sites at the mouths of the Rib, Gumara, and Abay rivers entering Lake Tana. The sampling locations were selected based on the assumption that these diverse environments may harbor distinct bacterial communities suitable for assessing bacterial diversity and comparing microbial distributions across different ecological zones [16].

Soil samples were collected from the upper 10–15 cm soil layer, where a large proportion of soil bacterial populations are concentrated, after removing approximately 3 cm of surface soil [17]. Except for sediment samples, all soil samples were collected using a sterile soil auger, transferred into sterile zip-lock polyethylene bags, and labeled immediately after collection. Sediment samples were collected at the sampling sites using an Ekman grab sampler. All samples were placed in an ice box and transported to the Postgraduate Microbiology Laboratory, where visible debris, pebbles, and other unwanted materials were removed. The samples were air-dried at 25 °C for 7–10 days [18], passed through a 2 mm sieve, and stored at 4 °C until further processing and microbiological analysis [19].

### 2.3 Isolation of bacterial isolates

One gram of each homogenized soil sample was suspended in 9 mL of sterile normal saline (0.85 % NaCl) and thoroughly mixed using a vortex mixer. Ten-fold serial dilutions were prepared from 10^−1^ to 10^−9^ using sterile normal saline (0.85 % NaCl), and aliquots from the appropriate dilution levels (10^−4^ to 10^−9^) were used for bacterial isolation. From each selected dilution, 0.1 mL of the suspension was spread onto nutrient agar plates in triplicate using a sterile L-shaped glass spreader (spread plate technique). The inoculated plates were incubated at 28 °C for 24–48 h. Following incubation, colonies exhibiting distinct morphological characteristics, including differences in size, shape, color, and texture, were selected and purified using the streak plate method. The purification process was repeated until pure bacterial cultures were obtained. Each purified isolate was maintained on nutrient agar slants at 4 °C for short-term storage and subcultured periodically for further analysis [20]. All culture media, glassware, and laboratory materials were sterilized by autoclaving at 121 °C for 15 min before use.

### 2.4 Identification and characterization of bacterial isolates

All purified isolates were examined microscopically to determine their cellular morphology, including cell size, shape, and arrangement. In addition, the Gram reaction of each isolate was determined using the Gram staining method. The Gram staining procedure was performed as follows: bacterial smears were heat-fixed, stained with crystal violet for 1 min, treated with Gram’s iodine for 1 min, decolorized with 95 % ethanol for 30 s, and counterstained with safranin for 1 min.

To evaluate the biochemical characteristics of the bacterial isolates, a series of biochemical tests were performed, including hydrogen sulfide (H_2_S) production using sulfide indole motility (SIM) medium, catalase activity, urease activity, starch hydrolysis, methyl red (MR) test, citrate utilization, indole production, and triple sugar iron (TSI) agar tests [21], [22], [23]. All biochemical tests were incubated at 37 °C for 24–48 h and the results were interpreted based on standard color changes, gas production, or other characteristic reaction patterns. Appropriate positive and negative controls were included where necessary to ensure the reliability of the tests. For example, *Staphylococcus aureus* was used as a positive control for the catalase test, whereas *Escherichia coli* were used as a negative control for the citrate utilization test.

### 2.5 Determination of physicochemical properties of soil

Fresh soil samples were air-dried and passed through a 2 mm sieve prior to physicochemical analysis. Soil pH was determined using a 1:2.5 (w/v) soil-to-distilled water ratio. The soil suspension was stirred for 5 min, and the pH was measured using a calibrated glass electrode pH meter according to the method described by [24]. Soil moisture content was determined by oven-drying 10 g of fresh soil at 105 °C until a constant weight was achieved (approximately 24 h). Soil texture was determined using the Bouyoucos hydrometer method on air-dried and sieved soil samples. A thermometer was used to measure the temperature of the soil suspension, and the required temperature correction was applied to the hydrometer readings according to standard procedures [25], 26].

Soil organic carbon content was determined using the Walkley–Black wet oxidation method [27]. Briefly, 1 g of air-dried and sieved soil was placed in a conical flask, followed by the addition of 10 mL of 1 N potassium dichromate. Subsequently, 20 mL of concentrated H_2_SO_4_ was carefully added, and the mixture was gently swirled and allowed to stand for 30 min. After dilution with distilled water and addition of ferroin indicator, the excess dichromate was titrated with 0.5 N ferrous ammonium sulfate until the color changed from violet/blue-green to green. The same procedure was performed using a reagent blank for correction.

Total nitrogen content was determined using the Kjeldahl digestion method [28]. Briefly, 0.5–1 g of soil sample was digested in a Kjeldahl flask containing the digestion mixture and concentrated H_2_SO_4_. The mixture was heated until a clear green solution was obtained, cooled, and diluted with distilled water. Sodium hydroxide (NaOH) solution was then added to release ammonia, which was distilled into a boric acid solution. The trapped ammonia was subsequently quantified by titration with standard hydrochloric acid (HCl) or sulfuric acid (H_2_SO_4_).

Available phosphorus was determined using the Olsen extraction method [29]. Briefly, 2.5 g of soil was extracted with 50 mL of 0.5 M sodium bicarbonate (NaHCO_3_) solution adjusted to pH 8.5 and shaken for 30 min. The extract was filtered, and an aliquot was used for phosphorus determination. The addition of ammonium molybdate and ascorbic acid reagents produced a blue-colored complex, and absorbance was measured using a spectrophotometer at wavelengths ranging from 660 to 882 nm. Phosphorus concentration was calculated using a standard calibration curve.

### 2.6 Data analysis

The raw data were compiled and organized using Microsoft Excel 2016. All statistical analyses were performed using R software version 4.3.2 [30]. Bacterial diversity among the isolates was estimated using the Shannon diversity index (H′) [31], which was calculated based on the relative abundance of distinct bacterial morphotypes. Principal component analysis (PCA) was conducted to visualize patterns, relationships, and clustering of bacterial isolates based on their biochemical characteristics [32]. Spearman’s rank correlation analysis was performed to assess the relationships between soil physicochemical properties and bacterial diversity across the 11 sampling sites [33]. For categorical variables, the chi-square test was applied to evaluate differences in frequency distributions, with a significance level set at *p* < 0.05. The results of all statistical analyses were presented using tables and figures.

## 3 Results

### 3.1 Morphological properties of isolates

Bacterial isolates were analyzed after removing duplicate entries. Among these isolates, 89 (85.6 %) were rod-shaped and 15 (14.4 %) were cocci, while 65 (62.5 %) were Gram-positive and 39 (37.5 %) were Gram-negative. Regarding motility characteristics, 89 (85.6 %) isolates were motile, whereas 15 (14.4 %) were non-motile. Of the total isolates, 53 (51.0 %) were non-spore-forming and 51 (49.0 %) were spore-forming. Colony surface characteristics revealed that 88 (84.6 %) isolates exhibited smooth surfaces, while 16 (15.4 %) showed rough surfaces. In terms of colony pigmentation, 26 (25.0 %) isolates produced creamy-colored colonies, whereas 78 (75.0 %) produced white colonies (Table 1).

**Table 1:** Morphological characterization of isolated bacteria.

Isolates	Colony morphology			
	Shape	GR	Motility	Spore
A1	Rod	_	+	_
A2	Rod	+	+	+
A3	Rod	+	+	+
A4	Rod	+	+	+
A5	Rod	+	+	+
A6	Rod	_	+	_
A7	Rod	_	+	_
A8	Rod	_	+	_
A9	Rod	_	+	_
A10	Rod	_	+	_
S1	Rod	_	+	_
S2	Rod	_	+	_
S3	Rod	_	_	_
S4	Cooci	+	_	_
S5	Rod	+	_	+
S6	Rod	+	+	+
S7	Rod	_	+	_
S8	Rod	+	+	+
S9	Cocci	+	_	_
S10	Rod	_	+	_
TF1	Cocci	+	_	_
TF2	Rod	_	+	_
TF3	Rod	_	+	_
TF4	Rod	_	+	_
TF5	Rod	+	+	+
TF6	Rod	_	+	_
TF7	Rod	_	+	_
TF8	Rod	+	+	+
TF9	Rod	_	+	_
TF10	Rod	+	+	+
TF11	Cocci	+	_	_
TF12	Rod	_	+	_
TF13	Cocci	+	_	_
TF14	Cocci	+	_	_
TI1	Rod	+	+	+
TI2	Rod	+	+	+
TI3	Rod	+	+	+
TI4	Rod	_	+	_
TI5	Rod	+	+	+
TI6	Cocci	+	_	_
TI7	Rod	_	+	_
TI8	Rod	+	+	+
TI9	Rod	_	+	_
TI10	Rod	_	+	_
TI11	Rod	+	+	+
TE1	Rod	+	+	+
TE2	Rod	+	+	+
TE3	Rod	+	+	+
TE4	Rod	+	+	+
TE5	Rod	_	+	_
TE6	Rod	+	+	+
TE7	Cocci	+	_	_
TE8	Rod	_	+	_
TE9	Rod	_	+	_
TO1	Rod	+	+	+
TO2	Rod	_	+	_
TO3	Rod	+	+	+
TO4	Rod	+	+	+
TO5	Rod	_	+	_
TO6	Rod	+	+	+
TO7	Rod	+	+	+
TO8	Rod	+	+	+
TO9	Rod	+	+	+
TO9	Rod	+	+	+
FI1	Rod	_	+	_
FI2	Rod	+	+	+
FI3	Cocci	+	_	_
FI4	Rod	+	+	+
FI5	Rod	+	+	+
FI6	Rod	_	+	_
FO1	Rod	_	+	_
FO2	Rod	+	+	+
FO3	Cocci	+	+	_
FO4	Rod	_	+	_
FO5	Rod	+	+	+
FO6	Rod	+	+	+
FO7	Cocci	+	+	_
R1	Rod	_	+	_
R2	Cocci	+	_	_
R3	Rod	+	+	+
R4	Rod	+	+	+
R5	Rod	+	+	+
R6	Cocci	+	_	_
R7	Rod	+	+	+
R8	Rod	+	+	+
R9	Rod	_	+	_
R10	Rod	+	+	+
AB1	Rod	_	+	_
AB2	Rod	_	+	_
AB3	Rod	+	+	+
AB4	Rod	+	+	+
AB5	Rod	_	+	_
AB6	Rod	_	+	_
AB7	Rod	_	+	_
AB8	Rod	+	+	+
AB9	Rod	+	+	+
AB10	Rod	+	+	+
AB11	Rod	+	+	+
AB12	Rod	+	+	+
GU1	Rod	+	+	+
GU2	Rod	_	+	_
GU3	Rod	+	+	+
GU4	Rod	+	+	+
GU5	Rod	+	+	+
GU6	Cocci	+	_	_
GU7	Cocci	+	_	_

**Figure 2:**
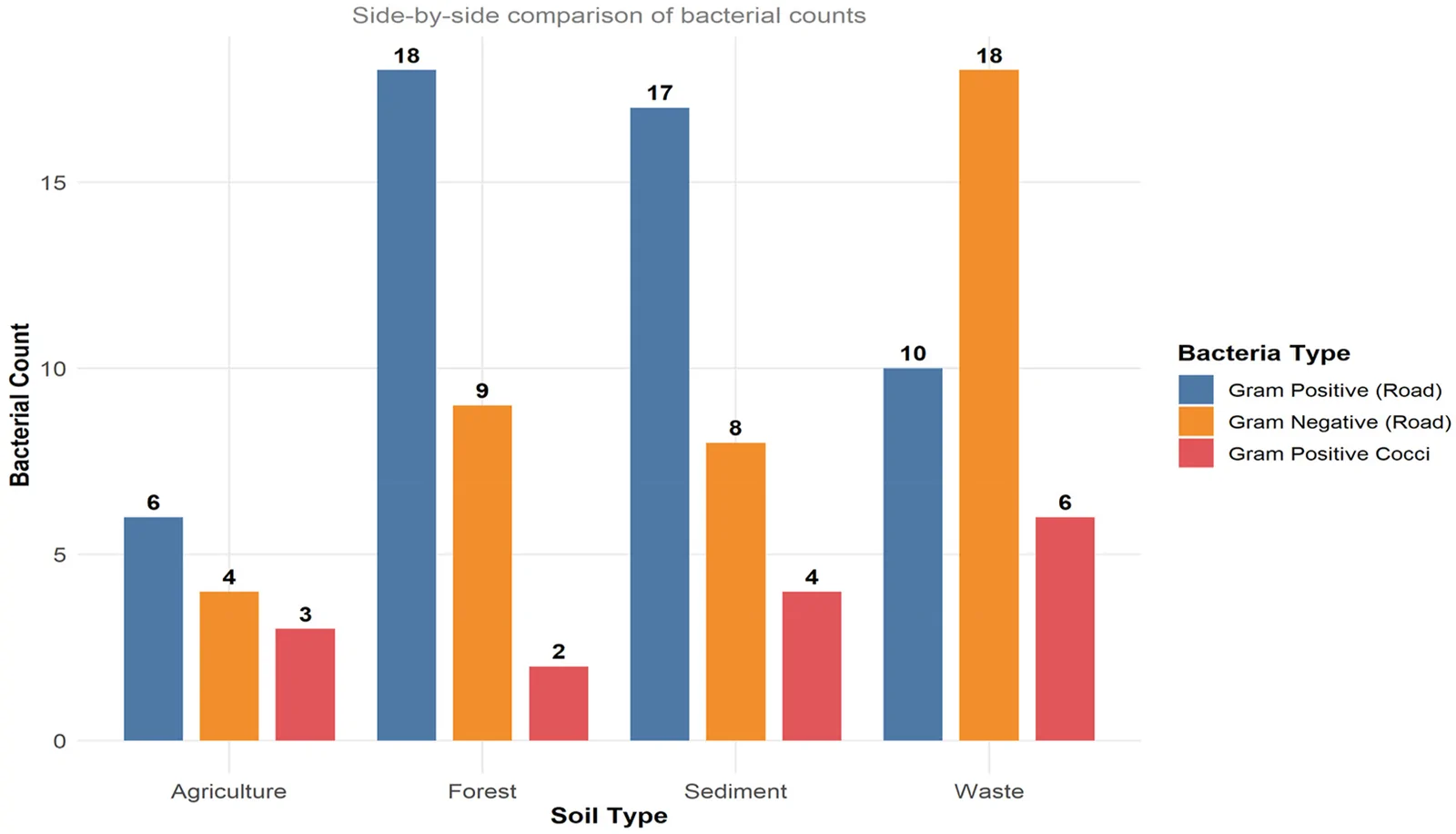
Distribution of Gram-positive rods, Gram-positive cocci, and Gram-negative rods across different soil types. Forest soils (TI, TE, TO) showed predominance of Gram-positive rods; waste soils (SW, AH, TF) showed higher proportions of Gram-negative rods; agricultural soil (FI, FO) showed balanced distribution; sediment soils (AB, RB, GU) showed predominance of Gram-positive rods.

Based on morphological characteristics, most isolates exhibited circular colonies with dry or mucoid textures. However, several morphological traits, including colony color, texture, and margin, exhibited considerable variation and served as useful discriminatory characteristics for differentiating among bacterial isolates. Based on Gram reaction and cellular morphology, Gram-positive rod-shaped bacteria were the most abundant group overall (48.6 %), followed by Gram-negative rod-shaped bacteria (37.1 %) and Gram-positive cocci (14.3 %) (Figure 2). Gram-positive rod-shaped isolates predominated in forest and sediment soils, whereas Gram-negative rod-shaped isolates were more frequently recovered from waste-impacted soils. Agricultural soils exhibited a relatively balanced distribution of Gram-positive and Gram-negative bacterial isolates. No Gram-negative cocci were detected among the bacterial isolates recovered from the sampled soils.

### 3.2 Biochemical properties of the isolates

The biochemical characteristics of the bacterial isolates revealed that 79 isolates were positive for sugar fermentation, 95 were catalase positive, 79 were methyl red positive, 65 were citrate positive, 32 were indole positive, 45 exhibited starch hydrolysis activity, 70 were urease positive, and 13 produced hydrogen sulfide (H_2_S) (Table 2).

**Table 2:** Biochemical characterization of isolated bacteria from each soil samples.

Isolate	Biochemical tests								Genus identification	Ref.
	Catalase	MR	Citrate	Indol	TSI	Starch	Urease	H2S		
A1	+	+	+	_	A/A + G	+	_	_	*Enterobacter*	[34]
A2	+	+	_	_	A/A	_	+	_	*Bacillus*	[35]
A3	+	+	_	_	A/A	_	_	_	*Bacillus*	[35]
A4	+	_	+	_	K/A	+	+	_	*Bacillus*	[35]
A5	+	_	+	_	K/K	_	+	_	*Bacillus*	[35]
A6	+	+	_	+	A/A + G	_	_	_	*Enterobacter*	[34]
A7	+	+	+	_	K/A	_	_	_	*Escherichia*	[36]
A8	_	+	_	_	K/K	_	_	_	*Pseudomonas*	[37]
A9	_	+	_	_	K/K	+	_	_	*Pseudomonas*	[37]
A10	_	_	+	_	K/K	+	_	_	*Pseudomonas*	[37]
S1	+	_	+	_	A/A	_	_	+	*Citrobacter*	[38]
S2	+	+	+	_	K/A	_	+	+	*Citrobacter*	[38]
S3	+	_	_	_	K/K	+	+	_	*Acinetobacter*	[39]
S4	_	+	_	_	A/A	_	+	_	*Enterococcus*	[40]
S5	+	+	+	+	A/A	+	_	_	*Bacillus*	[35]
S6	+	_	+	_	K/K	+	_	_	*Bacillus*	[35]
S7	+	+	+	+	K/A	_	_	_	*Escherichia*	[36]
S8	+	_	+	_	K/A	+	+	_	*Bacillus*	[35]
S9	_	+	_	_	K/A	_	+	_	*Enterococcus*	[40]
S10	+	_	+	_	A/A + G	+	+	+	*Proteus*	[41]
TF1	+	+	_	_	A/A	_	+	_	*Staphylococcus*	[42]
TF2	+	+	_	_	K/K	_	+	_	*Pseudomonas*	[37]
TF3	+	+	+	_	K/A	+	+	+	*Citrobacter*	[38]
TF4	+	_	+	_	A/A	_	_	_	*Enterobacter*	[34]
TF5	+	+	_	_	K/K	_	+	_	*Bacillus*	[35]
TF6	+	_	+	_	K/A	_	+	_	*Enterobacter*	[34]
TF7	+	+	_	+	K/A	+	_	+	*Escherichia*	[36]
TF8	+	+	_	_	K/A	_	+	_	*Bacillus*	[35]
TF9	+	+	_	+	A/A	+	+	_	*Escherichia*	[36]
TF10	+	+	_	+	A/A	+	+	_	*Bacillus*	[35]
TF11	+	+	_	_	A/A	_	+	_	*Staphylococcus*	[42]
TF12	+	+	+	_	K/A + G	_	+	_	*Enterobacter*	[34]
TF13	+	+	+	_	A/A	+	+	_	*Staphylococcus*	[42]
TF14	+	_	+	_	K/A	+	+	_	*Staphylococcus*	[42]
TI1	+	_	+	+	K/A	+	+	_	*Bacillus*	[35]
TI2	+	_	+	+	A/A	+	_	+	*Bacillus*	[35]
TI3	+	+	+	_	K/A	_	_	_	*Bacillus*	[35]
TI4	+	+	+	+	A/A + G	_	+	+	*Proteus*	[41]
TI5	+	_	_	+	A/A	_	+	_	*Bacillus*	[35]
TI6	+	+	_	_	K/A	_	+	_	*Staphylococcus*	[42]
TI7	+	_	+	+	A/A + G	+	+	+	*Proteus*	[41]
TI8	+	+	_	_	K/A	_	+	_	*Bacillus*	[35]
TI9	+	_	+	_	A/A	_	+	+	*Proteus*	[41]
TI10	+	+	_	+	K/A	_	_	_	*Escherichia*	[36]
TI11	+	+	+	_	K/K	+	+	_	*Bacillus*	[35]
TE1	+	+	+	+	A/A	+	+	_	*Bacillus*	[35]
TE2	+	+	+	_	K/A	_	+	_	*Bacillus*	[35]
TE3	+	+	_	_	K/A	+	+	_	*Bacillus*	[35]
TE4	+	+	_	_	K/A	+	_	+	*Bacillus*	[35]
TE5	+	+	_	_	K/K	_	_	_	*Pseudomonas*	[37]
TE6	+	+	+	_	A/A	_	+	_	*Bacillus*	[35]
TE7	+	+	+	_	A/A	_	+	_	*Staphylococcus*	[42]
TE8	+	_	+	_	K/K	_	+	_	*Pseudomonas*	[37]
TE9	_	_	+	_	A/A + G	+	+	+	*Proteus*	[41]
TO1	+	+	+	_	K/A	+	_	_	*Bacillus*	[35]
TO2	+	_	+	_	K/K	_	_	_	*Pseudomonas*	[37]
TO3	+	+	+	+	K/A	_	+	_	*Bacillus*	[35]
TO4	+	+	_	_	K/K	_	+	_	*Bacillus*	[35]
TO5	+	+	+	_	A/A	+	_	_	*Escherichia*	[36]
TO6	+	+	_	_	K/A	_	+	_	*Bacillus*	[35]
TO7	+	+	+	+	A/A	+	+	_	*Bacillus*	[35]
TO8	+	+	+	_	K/A	+	+	_	*Bacillus*	[35]
TO9	+	+	_	+	K/A	_	+	_	*Bacillus*	[35]
FI1	+	_	+	_	K/K	_	_	_	*Pseudomonas*	[37]
FI2	+	+	+	+	K/A	+	+	_	*Bacillus*	[35]
FI3	_	+	_	_	A/A	_	_	_	*Enterococcus*	[40]
FI4	+	+	_	_	A/A	+	+	_	*Bacillus*	[35]
FI5	+	+	_	+	A/A	_	+	_	*Bacillus*	[35]
FI6	+	_	+	_	K/K	_	+	_	*Pseudomonas*	[37]
FO1	+	_	+	_	K/K	_	+	_	*Pseudomonas*	[37]
FO2	+	+	+	+	A/A	+	+	_	*Bacillus*	[35]
FO3	+	+	+	_	K/A	+	+	_	*Staphylococcus*	[42]
FO4	+	+	_	+	K/A	_	+	_	*Escherichia*	[36]
FO5	+	+	_	_	K/A	+	_	_	*Bacillus*	[35]
FO6	+	_	+	_	K/A	+	+	_	*Bacillus*	[35]
FO7	+	+	+	_	K/A	+	+	_	*Staphylococcus*	[42]
R1	+	+	+	+	K/K	_	+	_	*Pseudomonas*	[37]
R2	+	+	+	_	K/A	_	+	_	*Staphylococcus*	[42]
R3	+	+	_	_	K/K	_	+	_	*Bacillus*	[35]
R4	+	+	+	_	K/A	+	+	_	*Bacillus*	[35]
R5	+	+	+	+	K/A	_	_	_	*Bacillus*	[35]
R6	+	+	+	_	K/A	+	_	_	*Staphylococcus*	[42]
R7	+	_	_	_	A/A	+	+	_	*Bacillus*	[35]
R8	+	+	_	+	K/A	_	_	_	*Bacillus*	[35]
R9	+	_	_	_	K/K	_	_	_	*Pseudomonas*	[37]
R10	+	+	+	_	K/A	+	_	_	*Bacillus*	[35]
AB1	+	+	+	+	K/A	_	_	_	*Escherichia*	[36]
AB2	+	+	+	+	K/A	_	_	_	*Escherichia*	[36]
AB3	+	+	+	+	A/A	_	+	_	*Bacillus*	[35]
AB4	+	+	_	+	K/A	_	+	_	*Bacillus*	[35]
AB5	+	_	+	_	K/A	_	+	+	*Proteus*	[41]
AB6	+	_	+	_	A/K	_	+	+	*Proteus*	[41]
AB7	+	+	+	_	K/A	_	+	+	*Citrobacter*	[38]
AB8	+	+	+	+	K/A	+	_	_	*Bacillus*	[35]
AB9	+	+	+	+	K/A	+	_	_	*Bacillus*	[35]
AB10	+	+	_	_	A/A	+	_	_	*Bacillus*	[35]
AB11	+	+	_	_	K/A	_	+	_	*Bacillus*	[35]
AB12	+	+	+	_	K/K	_	+	_	*Bacillus*	[35]
GU1	+	+	+	_	A/A	+	+	_	*Bacillus*	[35]
GU2	+	+	+	+	A/A	_	+	_	*Escherichia*	[36]
GU3	+	+	+	_	K/A	+	+	_	*Bacillus*	[35]
GU4	+	+	+	+	A/A	+	+	_	*Bacillus*	[35]
GU5	+	+	+	+	A/A	+	_	_	*Bacillus*	[35]
GU6	_	+	_	_	K/A	_	+	_	*Enterococcus*	[40]
GU7	_	+	_	_	A/A	_	_	_	*Enterococcus*	[40]

A: animal husbandry; S: solid waste; TF: tannery effluent; TI: Tara Gedam inner; TE: Tara Gedam edge; TO: Tara Gedam outer; FI: Fogera inner; FO: Fogera outer; AB: Abay river mouth; R: Rib river mouth; GU: Gumara river mouth; TSI: triple sugar iron agar; A/A + G: produce both glucose and lactose with Gas; A/K: produce only glucose; K/A: produce only lactose; K/K: not produce both.

**Figure 3:**
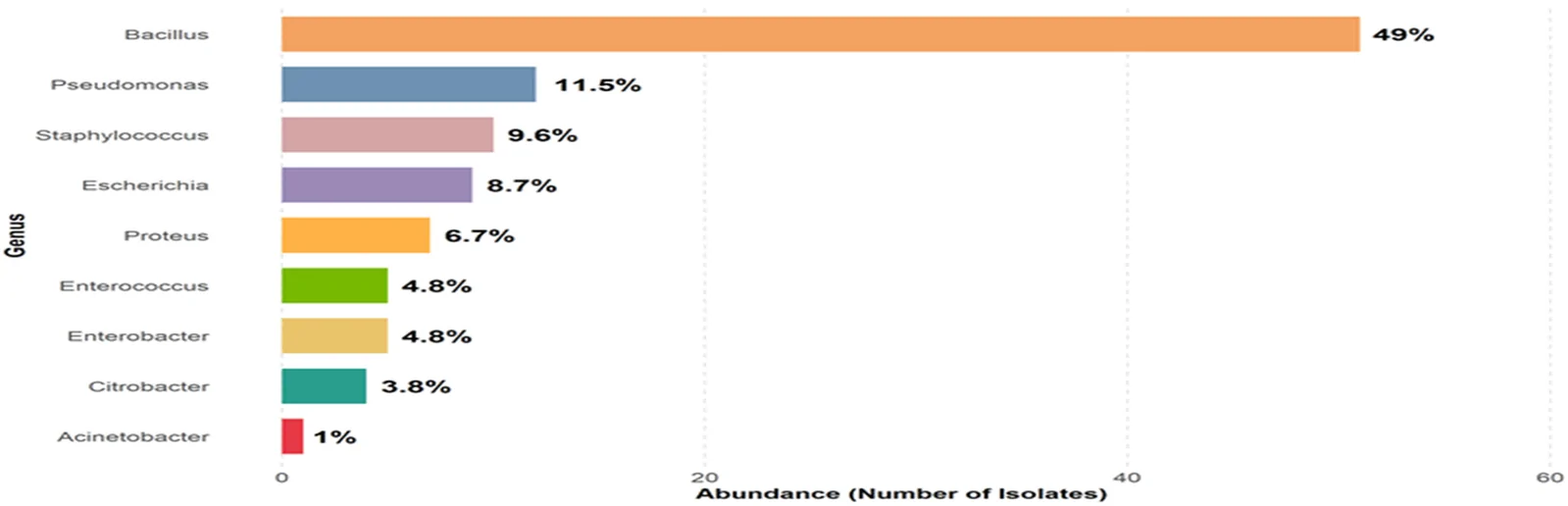
Abundance of bacterial genera among the 104 isolates. *Bacillus* dominated (49 %), followed by *Pseudomonas* (11.5 %), *Staphylococcus* (9.6 %), *Escherichia* (8.7 %), *Proteus* (6.7 %), *Enterococcus* (4.8 %), *Enterobacter* (4.8 %), *Citrobacter* (3.8 %), and *Acinetobacter* (1 %).

Based on their morphological and biochemical characteristics, the 104 bacterial isolates were tentatively classified into nine bacterial genera. The identified genera included *Bacillus*, *Pseudomonas*, *Staphylococcus*, *Escherichia*, *Proteus*, *Enterococcus*, *Enterobacter*, *Citrobacter*, and *Acinetobacter*. Among these, *Bacillus* was the predominant genus, accounting for 49.0 % of the isolates, followed by *Pseudomonas* (11.5 %) and *Staphylococcus* (9.6 %). The remaining genera included *Escherichia* (8.7 %), *Proteus* (6.7 %), *Enterococcus* and *Enterobacter* (4.8 % each), *Citrobacter* (3.8 %), and *Acinetobacter* (1.0 %). These findings indicate that *Bacillus* was the most frequently recovered genus among the culturable bacterial isolates, followed by *Pseudomonas* and *Staphylococcus* (Figure 3).

### 3.3 Physicochemical properties of soil

The soil samples analyzed in this study were collected from different ecological environments, including forest, agricultural cultivation, municipal solid waste, animal husbandry, tannery effluent, and sediment sites. A total of 11 soil samples were evaluated for physicochemical properties, including pH, moisture content, temperature, organic carbon (OC), total nitrogen (TN), and available phosphorus (AvP) (Table 3).

**Table 3:** Soil physicochemical properties of the study area (*n* = 11).

Soil properties	AH	SW	TF	TI	TE	TO	FI	FO	AB	RB	GU
PH	7.6	7.7	6.9	7.2	7.0	6.9	6.4	7.3	6.1	5.9	6.3
OC (%)	6.65	7.01	7.09	5.43	5.04	3.24	1.85	1.54	3.20	4.01	3.70
TN (%)	0.62	0.65	0.66	0.52	0.48	0.33	0.21	0.18	0.11	0.09	0.10
AvPPM	23.27	28.63	36.98	9.17	15.08	16.49	23.43	20.20	12.05	11.98	12.01
MC	19.13	9.10	18.0	16.83	15.22	8.67	7.50	6.50	72.5	70.1	70.79
Tm	23.76	23.80	23.80	23.33	23.70	23.80	22.96	22.90	24.1	23.8	24.0

TI: Tara Gedam inner; TE: Tara Gedam edge; TO: Tara Gedam outer; FI: Fogera inner; FO: Fogera outer; SW: municipal solid waste; AH: animal husbandry; TF: tannery effluent; AB: Abay; RB: Rib; GU: Gumara; OC: organic carbon; TN: total nitrogen; MC: moisture content; Tm: temperature; AvPPM: available phosphorus (parts per million).

The soil pH values across the 11 sampling sites ranged from 5.9 to 7.7, with a mean value of 6.8 ± 0.6. Two sites, Rib (RB; pH 5.9) and Gumara (GU; pH 6.3), exhibited acidic to slightly acidic conditions, whereas the remaining sites (AH, SW, TF, TI, TE, TO, FI, and FO) showed neutral to slightly alkaline conditions (pH 6.4–7.7).

Soil organic carbon (OC) content varied considerably among the sampling sites, ranging from 1.54 % at FO to 7.09 % at TF, with an overall mean value of 4.16 ± 1.84 %. Similarly, total nitrogen (TN) content ranged from 0.09 % at RB to 0.66 % at TF, with a mean value of 0.33 ± 0.21 %. Available phosphorus (AvP) showed substantial variation among the investigated soil properties, ranging from 9.17 ppm at TI to 36.98 ppm at TF, with a mean value of 18.94 ± 8.39 ppm.

Soil moisture content exhibited a distinct distribution pattern among sampling sites. The sediment-associated sites (AB, RB, and GU) showed exceptionally high moisture levels (70–73 %), whereas five sites (AH, SW, TF, TI, and TE) had moderate moisture contents (15–19 %). The remaining sites (TO, FI, and FO) exhibited relatively low moisture contents, ranging from 6.5 % to 8.7 %.

The study’s descriptive statistics revealed that temperature showed little variation (CV = 1.6 %), while moisture content had the highest coefficient of variation (96.9 %), indicating extreme geographical heterogeneity (Table 4).

**Table 4:** Descriptive statistics of soil physicochemical properties.

Soil property	Mean	SD	Min	Max	CV (%)
pH	6.90	0.600606	5.9	7.7	8.773789
Organic carbon (%)	4.432727	1.966703	1.54	7.09	44.36779
Total nitrogen (%)	0.359091	0.232485	0.09	0.66	64.74255
Available phosphorus (ppm)	19.02636	8.485552	9.17	36.98	44.59891
Moisture content (%)	28.57636	27.67693	6.5	72.5	96.85252
Temperature (°C)	23.63182	0.395849	22.9	24.1	1.675068

OC = organic carbon; TN = total nitrogen; MC = moisture content; Tm = temperature; AvPPM = available phosphorus.

The relationships between soil physicochemical properties and bacterial diversity across the 11 sampling sites were evaluated using Spearman’s rank correlation analysis. Soil pH (*ρ* = 0.688, *P* = 0.019), total nitrogen (TN) (*ρ* = 0.709, *P* = 0.015), and available phosphorus (AvP) (*ρ* = 0.645, *P* = 0.032) showed significant positive correlations with the Shannon diversity index (H′). Organic carbon (OC) exhibited a moderate positive correlation with Shannon diversity (*ρ* = 0.536); however, this relationship was not statistically significant (*P* = 0.089). No significant correlations were observed between Shannon diversity and either soil temperature or moisture content (*P* > 0.05).

Similarly, Simpson’s diversity index (1 − D) showed significant positive correlations with soil pH (*ρ* = 0.670, *P* = 0.024), total nitrogen (TN) (*ρ* = 0.700, *P* = 0.016), and available phosphorus (AvP) (*ρ* = 0.764, *P* = 0.006). Soil temperature and moisture content exhibited weak negative correlations with Simpson’s diversity index; however, these relationships were not statistically significant (*P* > 0.05). Organic carbon (OC) showed a moderate positive correlation with Simpson’s diversity index (*ρ* = 0.473), but this relationship was also not statistically significant (*P* = 0.142) (Table 5).

**Table 5:** Spearman’s rank correlation between soil properties and bacterial diversity indices (*n* = 11).

Soil property	Shannon H′(*ρ*)	*P*-value	Simpson (1 − D) (*ρ*)	*P*-value
pH	0.688	0.019	0.670	0.024
Organic carbon (%)	0.536	0.089	0.473	0.142
Total nitrogen (%)	0.709	0.015	0.700	0.016
Available phosphorus (ppm)	0.645	0.032	0.764	0.006
Moisture content (%)	−0.109	0.750	−0.227	0.502
Temperature (°C)	−0.149	0.662	−0.242	0.474

### 3.4 Diversity indices

The culturable bacterial community exhibited moderate diversity, with a Shannon diversity index (H′) of 1.68 and an evenness value of 0.72, indicating a relatively balanced distribution of bacterial genera. The effective number of equally abundant genera (Hill number, exp (H′)) was 3.6, suggesting that the observed diversity was equivalent to approximately four equally abundant genera. The presence of a low-abundance genus (11.1 % relative abundance) indicates variation in genus distribution and suggests that additional sampling or molecular approaches may reveal further bacterial diversity (Table 6).

**Table 6:** Diversity index summary.

Index	Value	Interpretation
Shannon diversity (H′)	1.679	Moderate diversity (95 % CI: 1.4496–2.1428)
Simpson diversity (1 − D)	0.7188	Moderate diversity (95 % CI: 0.6405–0.8784)
Pielou’s evenness (J′)	0.7641	High evenness
Simpson reciprocal (1/D)	3.56	Effective number of equally abundant genera = 3.6

**Figure 4:**
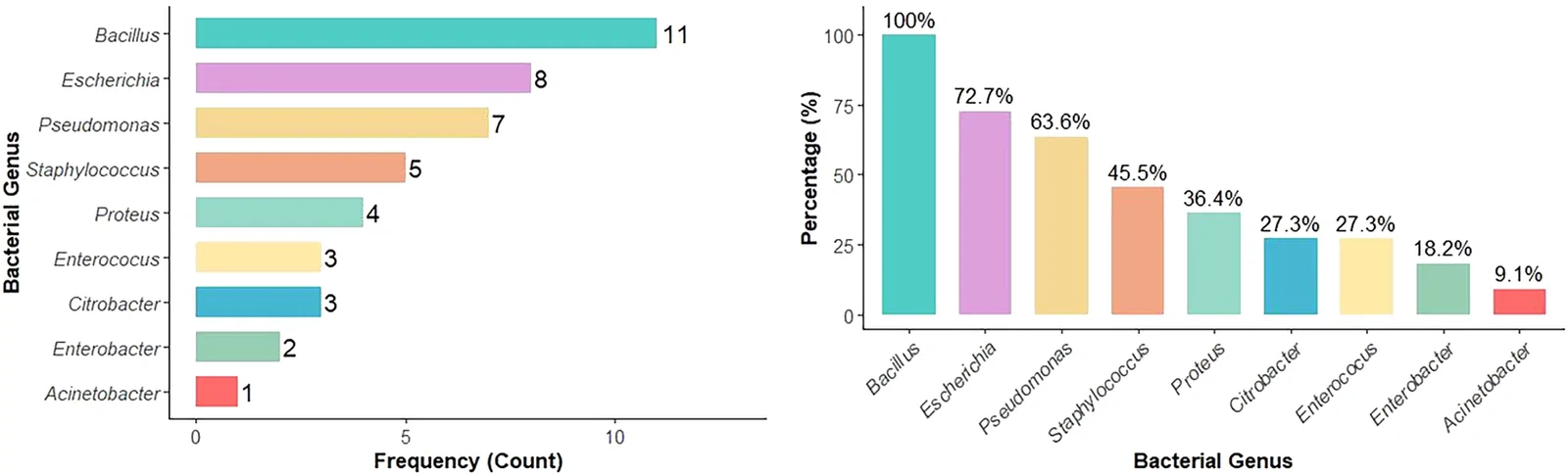
Frequency (left) and percentage (right) of bacterial genera across all sampling sites. *Bacillus* showed 100 % occurrence, followed *by Escherichia* (72.7 %) *and Pseudomonas* (63.6 %).

The distribution analysis revealed a hierarchical pattern of occurrence among the identified bacterial genera, with *Bacillus* spp. being the most frequently detected genus and occurring across all sampling sites (100 % occurrence). The high prevalence of *Bacillus* spp. suggests that members of this genus are widely adapted to the diverse environmental conditions represented in the study area. *Escherichia* spp. (72.7 %) and *Pseudomonas* spp. (63.6 %) were also frequently detected, indicating their broad distribution among the sampled environments. In contrast, less frequently detected genera, such as *Acinetobacter* spp., showed wider confidence intervals (1.6–37.7 %), reflecting greater uncertainty in their estimated occurrence due to their lower detection frequency. These findings suggest that additional sampling may provide further insights into the distribution patterns of less abundant culturable bacterial genera (Figure 4).

The chi-square test revealed a highly significant difference between the observed and expected frequencies of bacterial genera (*χ* ^2^ = 32.45, d*f* = 8, *p* < 0.001), indicating that the distribution of genera among the sampling sites was unlikely to have occurred by chance alone. This significant variation suggests that certain genera were more frequently recovered than others across the studied environments, possibly reflecting differences in environmental conditions and ecological preferences. The observed distribution pattern may indicate habitat-associated variation in culturable bacterial communities (Table 7).

**Table 7:** Chi-square test of bacteria.

Parameter	Value
Chi-square statistic	32.45
Degrees of freedom	8
***P*-value**	**<0.001**

The bold value indicates it has a strong significance values between the observed and expected frequencies of bacterial genera.

### 3.5 Principal component analysis

The morphological and biochemical characterization revealed that catalase activity, TSI reactions, motility, and Gram reaction showed high positive frequencies across all sampling sites. Similarly, methyl red, urease, citrate utilization, and spore formation were positive in a large proportion of isolates from most study sites. In contrast, indole production and hydrogen sulfide (H_2_S) production occurred less frequently, whereas starch hydrolysis showed an intermediate level of activity among the tested isolates.

**Figure 5:**
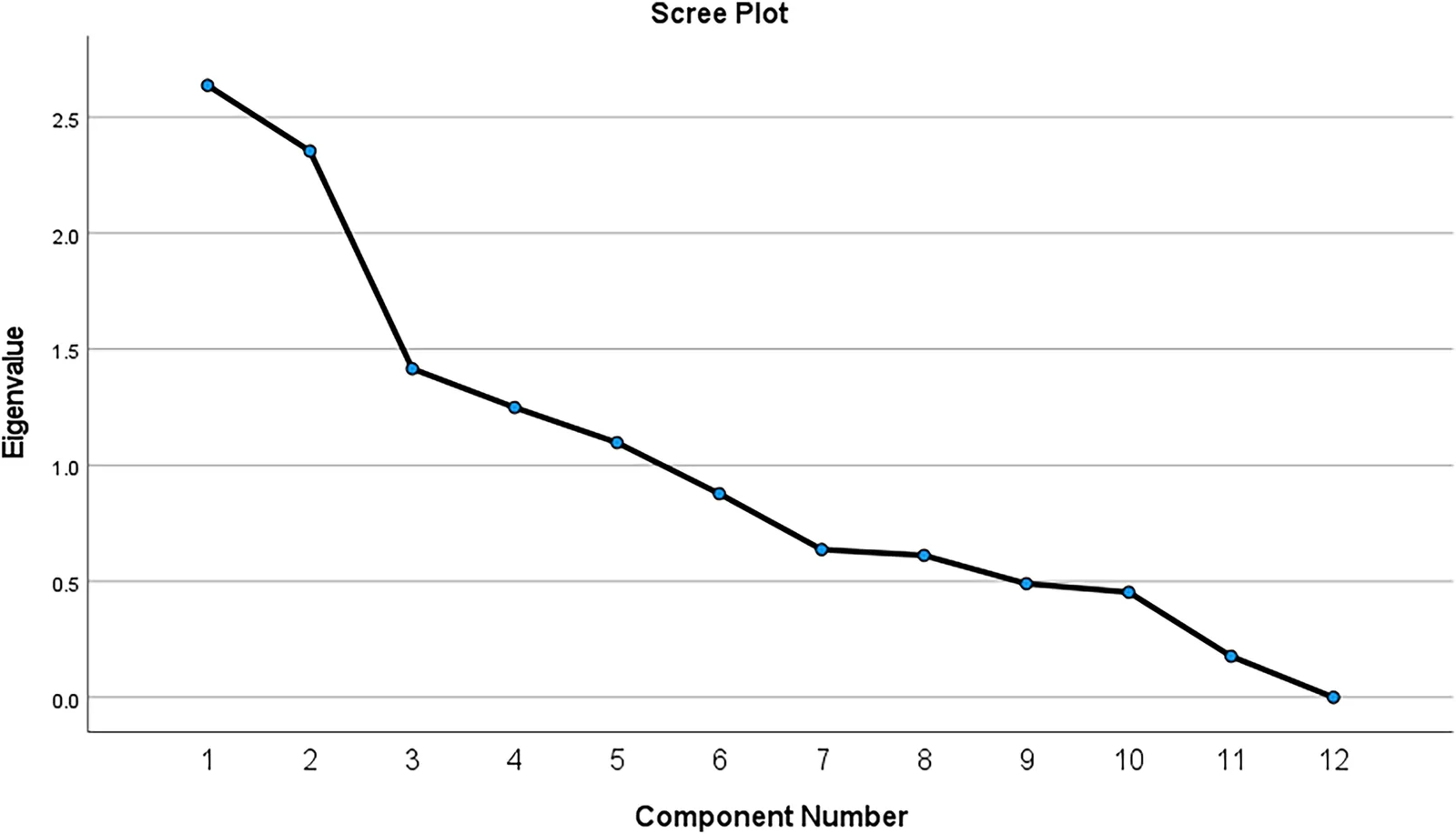
Principal component analysis (PCA) biplot showing the distribution of isolates based on morphological and biochemical characteristics. The first two principal components explain 55.4 % of the total variance. Vectors indicate the contribution of each test variable to the principal components.

Principal component analysis (PCA) was performed to reduce the dimensionality of the biochemical characterization dataset and identify the major variables contributing to variation among bacterial isolates. The first five principal components, each with an eigenvalue greater than one, explained approximately 73 % of the total variance (Figure 5; Table 8). The scree plot demonstrated a clear inflection point after the fifth component, supporting the retention of five principal components for interpretation.

**Table 8:** Variable loadings on principal components.

Component	Eigenvalue	% Variance	Key variables (loading)
PC1	4.82	34.4	Gram (0.82), motility (0.76), glucose (0.72), catalase (0.70), lactose (0.69)
PC2	2.94	21.0	Urease (0.68), indole (0.65), spore (0.60)
PC3	1.67	11.9	Citrate (0.66), oxidase (0.64)

The first principal component was strongly associated with Gram reaction (loading = 0.82) and motility (loading = 0.76), indicating that these characteristics contributed substantially to differentiation among the bacterial isolates. The second component was mainly influenced by catalase activity (loading = 0.70) and urease activity (loading = 0.68). The third component was associated primarily with indole production (loading = 0.65) and spore formation (loading = 0.60). The fourth component showed high contributions from sugar fermentation characteristics, including glucose fermentation (loading = 0.72) and lactose fermentation (loading = 0.69), while the fifth component was mainly explained by citrate utilization (loading = 0.66) and oxidase activity (loading = 0.64) (Table 8).

## 4 Discussion

A total of 104 culturable bacterial isolates representing nine genera were identified across all sampling sites. In the present study, the diversity of culturable bacterial isolates varied considerably among soil types, with waste-impacted soils exhibiting the highest Shannon and Simpson diversity indices. This increased diversity may be associated with the heterogeneous environmental conditions, nutrient inputs, and selective pressures present in waste-affected habitats. Similar observations have been reported by [43], who suggested that disturbed soils may support diverse bacterial communities due to enhanced nutrient availability and microbial adaptation to environmental stress.

The observed distribution pattern was also consistent with findings reported by [44], who documented the predominance of Gram-positive rod-shaped bacteria in forest and sediment soils, suggesting their adaptation to relatively stable environments with high organic matter availability. Furthermore [45], reported that agricultural management practices can reduce microbial diversity by promoting specific microbial groups, which agrees with the comparatively lower bacterial diversity observed in agricultural soils in the present study.

The diversity indices obtained in this study (Shannon diversity index, H′ = 1.68; Simpson’s diversity index, 1 − D = 0.72) indicated a moderate level of culturable bacterial diversity with a relatively even distribution among bacterial taxa. These findings are consistent with previous studies conducted under comparable agricultural soil conditions. Similar diversity values were reported by [46] in agricultural soils of Saraburi, Thailand, where the Simpson’s diversity index was 0.74 and the Shannon diversity index was 1.77. The similarity between these findings suggests that moderate bacterial diversity may be characteristic of agricultural ecosystems influenced by human activities, where repeated management practices, such as tillage and fertilizer application, may shape microbial communities by maintaining a balance between microbial richness and environmental selection pressures.

The predominance of *Bacillus* spp. (49 %) across the sampling sites is noteworthy and is consistent with previous studies investigating soil bacterial communities [10], 22]. Members of the genus *Bacillus* are well adapted to soil environments due to their ability to form endospores, which provide resistance to desiccation, heat, and other environmental stresses. In addition, their metabolic versatility, including the production of extracellular enzymes and diverse secondary metabolites with antimicrobial activity, contributes to their ecological success in various soil habitats [47]. The detection of *Bacillus* spp. across all sampling sites (100 % occurrence) indicates that this genus was the most widely distributed culturable bacterial group in the studied environments. This widespread distribution highlights the ecological adaptability of *Bacillus* spp. and their potential importance as target organisms for further investigation of antimicrobial compound production.

The high prevalence of *Bacillus* spp. and *P.* spp. in waste-impacted soils (SW, AH, and TF) is particularly relevant for bioprospecting, as members of these genera are widely recognized producers of antimicrobial compounds and other bioactive secondary metabolites [10], 12]. The occurrence of these genera in tannery effluent and municipal solid waste environments suggests that they may possess adaptive traits that enable survival under contaminated conditions, including possible tolerance to heavy metals and other environmental stresses [11]. Therefore, these isolates represent promising candidates for further investigation of antimicrobial activity and potential biotechnological applications, including bioremediation.

The culturable soil bacterial community demonstrated considerable metabolic versatility, as indicated by the high proportion of sugar-fermenting isolates (75.9 %) and catalase-positive isolates (91 %). These traits suggest that many of the recovered bacteria possess diverse metabolic capabilities that may facilitate survival under changing environmental conditions. Similar observations were reported by [48], who noted that soil bacteria exposed to reactive oxygen species frequently produce catalase as a protective physiological response. Likewise [49], highlighted that catalase activity and carbohydrate utilization are important physiological characteristics that enhance bacterial survival and ecological fitness in terrestrial environments.

The ability of bacteria to utilize and ferment a range of carbohydrate substrates enables them to exploit diverse organic carbon sources and may contribute to organic matter decomposition and carbon cycling in soil ecosystems. Furthermore, catalase activity provides protection against oxidative stress by converting hydrogen peroxide into water and oxygen, thereby supporting the survival of aerobic and facultative anaerobic bacteria in oxygen-exposed soil environments.

A considerable proportion of the culturable bacterial isolates demonstrated starch-hydrolyzing activity (43.2 %), indicating their potential ability to produce extracellular amylases involved in the degradation of complex polysaccharides. Similar findings were reported by [50], who identified extracellular enzyme production, including amylases, as an important mechanism through which soil bacteria contribute to the decomposition of organic matter. Likewise [51], emphasized the essential role of microbial extracellular enzymes in regulating carbon turnover and supporting ecosystem processes, which is consistent with the observations of the present study.

In contrast, indole production was detected in only 30.7 % of the isolates, while hydrogen sulfide (H_2_S) production was observed in 12.5 % of isolates, suggesting that these metabolic traits were less frequently distributed among the recovered bacterial groups. Similar patterns were reported by [6], who noted that indole and H_2_S production are generally restricted to specific bacterial taxa and may occur at lower frequencies depending on environmental conditions and microbial community composition.

Principal component analysis (PCA) revealed that Gram reaction and motility contributed most strongly to the first principal component, indicating that these characteristics were important variables for differentiating the phenotypic profiles of the bacterial isolates. Differences in cell wall structure between Gram-positive and Gram-negative bacteria influence various physiological properties, including environmental adaptation, nutrient acquisition, and tolerance to external stresses. Similarly, bacterial motility may enhance the ability of microorganisms to colonize heterogeneous soil microsites and respond to favorable nutrient gradients.

Previous studies have reported that bacterial functional traits, including cell wall characteristics and physiological adaptations, contribute significantly to ecological strategies and community composition in soil environments [52]. Furthermore [53], emphasized that motility represents an important adaptive trait that enables soil bacteria to respond to environmental fluctuations and changes in nutrient availability. Overall, the PCA findings suggest that the observed organization of culturable bacterial isolates was associated with both structural characteristics (Gram reaction and motility) and metabolic traits (enzyme activities and substrate utilization patterns).

The present study demonstrated that culturable bacterial diversity was significantly and positively correlated with soil pH, total nitrogen (TN), and available phosphorus (AP). In contrast, organic carbon (OC), soil moisture content, and soil temperature showed no significant correlations with bacterial diversity. These findings are consistent with numerous studies indicating that soil pH is one of the most important environmental factors shaping bacterial community composition and diversity across diverse ecosystems.

At a continental scale in North American ecosystems [54], reported a strong relationship between soil pH and bacterial diversity, highlighting the importance of pH as a major determinant of bacterial community patterns. Similarly [55], demonstrated that soil pH explained greater variation in bacterial community structure than other measured soil properties across different land-use systems. Furthermore [56], reported that acidic soil conditions can restrict the distribution of many bacterial groups, whereas bacterial diversity generally increases as soil pH approaches neutral conditions.

Additionally, total nitrogen (TN) showed a significant positive correlation with both the Shannon and Simpson diversity indices. Higher nitrogen availability may reduce nutrient limitations and support greater bacterial biomass and diversity by providing essential resources for microbial growth. Similar findings were reported by [2], who identified soil nutrient availability, particularly nitrogen, as an important factor influencing microbial diversity across global ecosystems. Likewise [57], demonstrated that increased soil nitrogen content in agricultural soils was associated with changes in bacterial community composition and enhanced bacterial diversity.

Available phosphorus (AP) showed the strongest positive correlation with Simpson’s diversity index, suggesting that phosphorus availability may play an important role in shaping the structure and evenness of culturable bacterial communities in the studied environments. These findings are consistent with reports by [58], who demonstrated that nutrient availability influences bacterial diversity across tropical ecosystems, and by [59], who found that phosphorus enrichment significantly affected soil bacterial community composition. Increased phosphorus availability may promote microbial growth and support the persistence of diverse bacterial groups by providing additional resources for metabolically distinct microorganisms.

Bacterial diversity showed a moderate positive correlation with soil organic carbon (OC); however, this relationship was not statistically significant. Since organic carbon represents an important energy source for heterotrophic microorganisms, higher OC availability is often expected to support greater microbial abundance and diversity. However, the absence of a significant correlation in the present study may be related to several factors, including the relatively small number of sampling sites (*n* = 11), spatial variability among sampling locations, and the influence of other environmental variables not considered in this analysis. Similar findings were reported by [60], who suggested that the relationship between soil organic carbon and bacterial diversity is often influenced by additional factors, including vegetation type, soil management practices, nutrient availability, and interactions among soil properties, rather than carbon content alone.

Neither soil moisture content nor temperature showed a significant correlation with culturable bacterial diversity in the present study. Although both factors are known to influence microbial activity and physiological processes, the limited variation in soil temperature during the sampling period and spatial differences in soil moisture among sampling sites may have reduced their detectable relationships with bacterial diversity. Similar observations were reported by [17], who found that short-term temperature fluctuations often have a weaker influence on bacterial community composition than soil chemical properties. Likewise [61], reported that soil moisture primarily affects temporal variations in microbial activity rather than long-term patterns of bacterial diversity.

Overall, the findings of the present study suggest that soil chemical properties, particularly pH, nitrogen, and phosphorus availability, were the major environmental variables associated with variations in culturable bacterial diversity across the studied habitats. These results agree with previous ecological studies indicating that soil chemical characteristics often play a stronger role in shaping bacterial diversity patterns than short-term variations in physical factors such as moisture and temperature.

## 5 Study limitations

This study has several limitations that should be acknowledged. First, bacterial identification was based exclusively on morphological and biochemical characterization. The application of molecular approaches, such as 16S rRNA gene sequencing, would provide more accurate taxonomic identification and allow confirmation of bacterial identities. These analyses, together with antimicrobial activity screening and secondary metabolite characterization, will be addressed in the subsequent objectives of this research.

Second, because sampling was conducted only during the dry season (March–June 2024), seasonal variations in bacterial abundance, diversity, and community composition may not have been captured. Third, the use of a single culture medium (nutrient agar) may have limited the recovery of bacteria with different nutritional requirements. Future studies should incorporate sampling across different seasons and employ multiple selective and differential media to improve the recovery and characterization of a broader range of culturable bacterial diversity.

## 6 Conclusions

This study characterized 104 culturable bacterial isolates recovered from 11 contrasting soil habitats in the Amhara region of Ethiopia. The findings indicate that soil pH, total nitrogen, and available phosphorus were the major environmental variables associated with variations in bacterial diversity. Gram-positive, rod-shaped, and catalase-positive bacteria predominated across the sampling sites, with *Bacillus* spp. being the most frequently recovered genus (49 %), based on morphological and biochemical characterization.

The diversity indices (Shannon diversity index, H′ = 1.68; Simpson’s diversity index, 1 − D = 0.72) indicated moderate culturable bacterial diversity with a relatively even distribution among bacterial genera. The frequent occurrence of *Bacillus* spp. and *P.* spp., which are recognized producers of diverse antimicrobial secondary metabolites, highlights the potential of these soil environments as valuable sources for future antimicrobial compound discovery. Furthermore, the high enzymatic activity and metabolic versatility observed among the isolates suggest their potential applications in biotechnology, including bioremediation, enzyme production, and agricultural biofertilization.

## Supplementary Material

Supplementary Material

Supplementary Material

## References

[1] Nannipieri P, Ascher-Jenull J, Ceccherini MT, Pietramellara G, Renella G, Schloter M (2020). Beyond microbial diversity for predicting soil functions: a mini review. Pedosphere.

[2] Delgado-Baquerizo M, Maestre FT, Reich PB, Jeffries TC, Gaitán JJ, Encinar D (2016). Microbial diversity drives multifunctionality in terrestrial ecosystems. Nat Commun.

[3] Liu D, Yang Y, An S, Wang H, Wang Y (2018). The biogeographical distribution of soil bacterial communities in the loess plateau as revealed by high-throughput sequencing. Front Microbiol.

[4] Chen Y, Wang X, Li Z (2024). Environmental factors shaping soil microbial communities. Environ Res.

[5] Bochner BR (2009). Global phenotypic characterization of bacteria. FEMS Microbiol Rev.

[6] Cappuccino JG, Welsh C (2017). Microbiology: a laboratory manual.

[7] Hafezi A, Khamar Z (2024). The method and analysis of some biochemical tests commonly used for microbial identification: a review. Compr Health Biomed Stud.

[8] Yang B, Pang X, Hu B, Bao W, Tian G (2017). Does thinning-induced gap size result in altered soil microbial community in pine plantation in eastern Tibetan Plateau?. Ecol Evol.

[9] Ahn JH, Song J, Kim BY, Kim MS, Joa JH, Weon HY (2012). Characterization of the bacterial and archaeal communities in rice field soils subjected to long-term fertilization practices. J Microbiol.

[10] Zhou Y, Wang X, Liu J (2020). Antibiotic-producing bacteria in landfill soils. J Hazard Mater.

[11] Mahmud S, Ahmed R, Rahman M (2024). Heavy metal resistance in bacteria from tannery waste. Bioresour Technol.

[12] Gufe C, Chari TA, Jambwa P, Dinginya L, Mahlangu P, Marowero ST (2025). A review on the occurrence of antibiotic-resistant bacteria found in abattoir effluent from resource-limited settings: the necessity of a robust One Health framework. Sustain Environ.

[13] Imhoff JF (2016). Natural products from marine bacteria – extremophiles and their potential applications. Mar Drugs.

[14] Yitaferu B (2011). Agricultural climatic zones of the Amhara region. Ethiop J Agric Sci.

[15] Molla E, Gebrekidan H, Mamo T, Assen M (2009). Effects of land use change on selected soil properties in the Tara Gedam catchment and adjacent agro-ecosystems, northwest Ethiopia. Ethiop J Nat Resour.

[16] Baker KL, Langenheder S, Nicol GW, Ricketts D, Killham K, Campbell CD (2009). Environmental and spatial characterisation of bacterial community composition in soil to inform sampling strategies. Soil Biol Biochem.

[17] Fierer N, Schimel JP, Holden PA (2003). Variations in microbial community composition through two soil depth profiles. Soil Biol Biochem.

[18] Ahmed SR, Roy R, Romi IJ, Hasan M, Bhuiyan MKH, Khan MMH (2019). Phytochemical screening, antioxidant and antibacterial activity of some medicinal plants grown in Sylhet region. IOSR J Pharm Biol Sci.

[19] Brooks JP, Yates MV, Nakatsu CH, Miller RV, Pillai SD (2016). Soil sampling for microbial analyses. Manual of environmental microbiology.

[20] Cappuccino JG, Sherman N (2014). Microbiology: a laboratory manual.

[21] Collins CH, Lyne PM, Grange JM (1989). Microbiological methods.

[22] Paulsen IT, Holmes AJ (2014). Environmental microbiology: methods and protocols.

[23] Zaved HK, Rahman MM, Rahman MM, Rahman A, Arafat SMY, Rahman MS (2008). Isolation and characterization of effective bacteria for solid waste degradation for organic manure. KMITL Sci Technol J.

[24] Mosley LM, Rengasamy P, Fitzpatrick RW (2024). Soil pH: techniques, challenges and insights from a global dataset. Eur J Soil Sci.

[25] Bouyoucos GJ (1962). Hydrometer method improved for making particle size analyses of soils. Agron J.

[26] Gee GW, Bauder JW (1979). Particle-size analysis by hydrometer: a simplified method for routine textural analysis and a sensitivity test of measurement parameters. Soil Sci Soc Am J.

[27] Jha P, Biswas AK, Lakaria BL, Saha R, Singh M, Rao AS (2014). Predicting total organic carbon content of soils from Walkley and Black analysis. Commun Soil Sci Plant Anal.

[28] McGeehan SL, Naylor DV (1988). Automated instrumental analysis of carbon and nitrogen in plant and soil samples. Commun Soil Sci Plant Anal.

[29] Olsen SR, Cole CV, Watanabe FS, Dean LA (1954). Estimation of available phosphorus in soils by extraction with sodium bicarbonate.

[30] R Core Team (2023). R: a language and environment for statistical computing.

[31] Shannon CE (1948). A mathematical theory of communication. Bell Syst Tech J.

[32] Jolliffe IT (2002). Principal component analysis.

[33] Spearman C (1904). The proof and measurement of association between two things. Am J Psychol.

[34] Kumari K, Kumar P, Shweta S, Rajnish S, Singh P (2023). Molecular characterization and in-depth genome analysis of Enterobacter sp. S-16. Funct Integr Genomics.

[35] Koilybayeva M, Shynykul Z, Ustenova G, Abzaliyeva S, Alimzhanova M, Amirkhanova A (2023). Molecular characterization of some *Bacillus* species from vegetables and evaluation of their antimicrobial and antibiotic potency. Molecules.

[36] Leimbach A, Hacker J, Dobrindt U (2013). *Escherichia coli* as an all-rounder: the thin line between commensalism and pathogenicity. Int J Med Microbiol.

[37] Peix A, Ramírez-Bahena MH, Velázquez E (2009). Historical evolution and current status of the taxonomy of genus Pseudomonas. Infect Genet Evol.

[38] Brenner DJ, Grimont PAD, Steigerwalt AG, Fanning GR, Ageron E, Riddle CF (1993). Classification of citrobacter species by DNA hybridization and biochemical characteristics. Int J Syst Bacteriol.

[39] Bergogne-Bérézin E, Towner KJ (1996). Acinetobacter spp. as nosocomial pathogens: microbiological, clinical, and epidemiological features. Clin Microbiol Rev.

[40] Facklam RR, Collins MD (1989). Identification of Enterococcus species isolated from human infections by a conventional test scheme. J Clin Microbiol.

[41] O’Hara CM, Brenner FW, Miller JM (2000). Classification, identification, and clinical significance of Proteus, Providencia, and Morganella. Clin Microbiol Rev.

[42] Bannerman TL (2003). Staphylococcus, Micrococcus, and other catalase-positive cocci that grow aerobically. Manual of clinical microbiology.

[43] Jangid K, Williams MA, Franzluebbers AJ, Sanderlin JS, Reeves JH, Jenkins MB (2008). Relative impacts of land-use, management intensity and fertilization upon soil microbial community structure in agricultural systems. Soil Biol Biochem.

[44] Andrews JH, Carroll GC (2001). Microbial communities in forest soils. Mycologia.

[45] Osburn ED, McBride SG, Strickland MS (2023). Agricultural management effects on soil bacterial diversity. Agric Ecosyst Environ.

[46] Pakdee P (2020). The relationships between microbial communities and environmental factors in agricultural soils in Saraburi [bachelor’s senior project].

[47] Fira D, Dimkić I, Berić T, Lozo J, Stanković S (2018). Biological control of plant pathogens by Bacillus species. J Biotechnol.

[48] Madigan MT, Bender KS, Buckley DH, Sattley WM, Stahl DA (2021). Brock biology of microorganisms.

[49] Atlas RM, Bartha R (1998). Microbial ecology: fundamentals and applications.

[50] Das SK, Varma A, Shukla G, Varma A (2011). Role of enzymes in maintaining soil health. Soil enzymology. Soil biology.

[51] Burns RG, DeForest JL, Marxsen J, Sinsabaugh RL, Stromberger ME, Wallenstein MD (2013). Soil enzymes in a changing environment: current knowledge and future directions. Soil Biol Biochem.

[52] Fierer N, Bradford MA, Jackson RB (2007). Toward an ecological classification of soil bacteria. Ecology.

[53] De Bruijn FJ (2015). Biological nitrogen fixation.

[54] Fierer N, Jackson RB (2006). The diversity and biogeography of soil bacterial communities. Proc Natl Acad Sci U S A.

[55] Lauber CL, Hamady M, Knight R, Fierer N (2009). Pyrosequencing-based assessment of soil pH as a predictor of soil bacterial community structure. Appl Environ Microbiol.

[56] Rousk J, Bååth E, Brookes PC, Lauber CL, Lozupone C, Caporaso JG (2010). Soil bacterial and fungal communities across a pH gradient in an arable soil. ISME J.

[57] Wang J, Shi X, Zheng C, Suter H, Huang Z (2021). Different responses of soil bacterial and fungal communities to nitrogen deposition in a subtropical forest. Sci Total Environ.

[58] Tripathi BM, Stegen JC, Kim M, Dong K, Adams JM, Lee YK (2018). Soil pH mediates the balance between stochastic and deterministic assembly of bacteria. ISME J.

[59] Leff JW, Jones SE, Prober SM, Barberán A, Borer ET, Firn JL (2015). Consistent responses of soil microbial communities to elevated nutrient inputs in grasslands worldwide. Proc Natl Acad Sci U S A.

[60] Fierer N, Leff JW, Adams BJ, Nielsen UN, Bates ST, Lauber CL (2012). Cross-biome metagenomic analyses of soil microbial communities. Proc Natl Acad Sci U S A.

[61] Brockett BFT, Prescott CE, Grayston SJ (2012). Soil moisture is the major factor influencing microbial community structure and enzyme activities across seven biogeoclimatic zones in western Canada. Soil Biol Biochem.

