# Supplementary material for: Morphological and biochemical characterization of bacterial isolates from diverse soil habitats in the Amhara region, Ethiopia
Source: Open Life Sci. 2026 Sep 25;21(1):20251379. doi: 10.1515/biol-2025-1379 (PMC13614831; doi:10.1515/biol-2025-1379)
Supplement: Supplementary file 1 [file j_biol-2025-1379_suppl_001.docx]

**Supplement Table 1:** Morphological characterization of isolated bacteria

| **Isolates** | **Colony Morphology** | | | |
| --- | --- | --- | --- | --- |
|  | **Shape** | **GR** | **Motility** | **Spore** |
| A1 | Rod | _ | + | _ |
| A2 | Rod | + | + | + |
| A3 | Rod | + | + | + |
| A4 | Rod | + | + | + |
| A5 | Rod | + | + | + |
| A6 | Rod | _ | + | _ |
| A7 | Rod | _ | + | _ |
| A8 | Rod | _ | + | _ |
| A9 | Rod | _ | + | _ |
| A10 | Rod | _ | + | _ |
| S1 | Rod | _ | + | _ |
| S2 | Rod | _ | + | _ |
| S3 | Rod | _ | _ | _ |
| S4 | Cooci | + | _ | _ |
| S5 | Rod | + | _ | + |
| S6 | Rod | + | + | + |
| S7 | Rod | _ | + | _ |
| S8 | Rod | + | + | + |
| S9 | Cocci | + | _ | _ |
| S10 | Rod | _ | + | _ |
| TF1 | Cocci | + | _ | _ |
| TF2 | Rod | _ | + | _ |
| TF3 | Rod | _ | + | _ |
| TF4 | Rod | _ | + | _ |
| TF5 | Rod | + | + | + |
| TF6 | Rod | _ | + | _ |
| TF7 | Rod | _ | + | _ |
| TF8 | Rod | + | + | + |
| TF9 | Rod | _ | + | _ |
| TF10 | Rod | + | + | + |
| TF11 | Cocci | + | _ | _ |
| TF12 | Rod | _ | + | _ |
| TF13 | Cocci | + | _ | _ |
| TF14 | Cocci | + | _ | _ |
| TI1 | Rod | + | + | + |
| TI2 | Rod | + | + | + |
| TI3 | Rod | + | + | + |
| TI4 | Rod | _ | + | _ |
| TI5 | Rod | + | + | + |
| TI6 | Cocci | + | _ | _ |
| TI7 | Rod | _ | + | _ |
| TI8 | Rod | + | + | + |
| TI9 | Rod | _ | + | _ |
| TI10 | Rod | _ | + | _ |
| TI11 | Rod | + | + | + |
| TE1 | Rod | + | + | + |
| TE2 | Rod | + | + | + |
| TE3 | Rod | + | + | + |
| TE4 | Rod | + | + | + |
| TE5 | Rod | _ | + | _ |
| TE6 | Rod | + | + | + |
| TE7 | Cocci | + | _ | _ |
| TE8 | Rod | _ | + | _ |
| TE9 | Rod | _ | + | _ |
| TO1 | Rod | + | + | + |
| TO2 | Rod | _ | + | _ |
| TO3 | Rod | + | + | + |
| TO4 | Rod | + | + | + |
| TO5 | Rod | _ | + | _ |
| TO6 | Rod | + | + | + |
| TO7 | Rod | + | + | + |
| TO8 | Rod | + | + | + |
| TO9 | Rod | + | + | + |
| TO9 | Rod | + | + | + |
| FI1 | Rod | _ | + | _ |
| FI2 | Rod | + | + | + |
| FI3 | Cocci | + | _ | _ |
| FI4 | Rod | + | + | + |
| FI5 | Rod | + | + | + |
| FI6 | Rod | _ | + | _ |
| FO1 | Rod | _ | + | _ |
| FO2 | Rod | + | + | + |
| FO3 | Cocci | + | + | _ |
| FO4 | Rod | _ | + | _ |
| FO5 | Rod | + | + | + |
| FO6 | Rod | + | + | + |
| FO7 | Cocci | + | + | _ |
| R1 | Rod | _ | + | _ |
| R2 | Cocci | + | _ | _ |
| R3 | Rod | + | + | + |
| R4 | Rod | + | + | + |
| R5 | Rod | + | + | + |
| R6 | Cocci | + | _ | _ |
| R7 | Rod | + | + | + |
| R8 | Rod | + | + | + |
| R9 | Rod | _ | + | _ |
| R10 | Rod | + | + | + |
| AB1 | Rod | _ | + | _ |
| AB2 | Rod | _ | + | _ |
| AB3 | Rod | + | + | + |
| AB4 | Rod | + | + | + |
| AB5 | Rod | _ | + | _ |
| AB6 | Rod | _ | + | _ |
| AB7 | Rod | _ | + | _ |
| AB8 | Rod | + | + | + |
| AB9 | Rod | + | + | + |
| AB10 | Rod | + | + | + |
| AB11 | Rod | + | + | + |
| AB12 | Rod | + | + | + |
| GU1 | Rod | + | + | + |
| GU2 | Rod | _ | + | _ |
| GU3 | Rod | + | + | + |
| GU4 | Rod | + | + | + |
| GU5 | Rod | + | + | + |
| GU6 | Cocci | + | _ | _ |
| GU7 | Cocci | + | _ | _ |
|  |  |  |  |  |
