# Supplementary material for: Morphological and biochemical characterization of bacterial isolates from diverse soil habitats in the Amhara region, Ethiopia
Source: Open Life Sci. 2026 Sep 25;21(1):20251379. doi: 10.1515/biol-2025-1379 (PMC13614831; doi:10.1515/biol-2025-1379)
Supplement: Supplementary file 2 [file j_biol-2025-1379_suppl_002.docx]

**Supplement** **Table 2****:** Biochemical characterization of isolated bacteria from each soil samples

| **Isolates** | **Biochemical Tests** | | | | | | | | |  |  |
| --- | --- | --- | --- | --- | --- | --- | --- | --- | --- | --- | --- |
|  | **Catalase** | **MR** | **Citrate** | **Indol** | **TSI** | **Starch** | **Urease** | **H2S** | **Genus Identification** | | **References** |
| A1 | + | + | + | _ | A/A+G | + | _ | _ | *Enterobacter* | | [34] |
| A2 | + | + | _ | _ | A/A | _ | + | _ | *Bacillus* | | [35] |
| A3 | + | + | _ | _ | A/A | _ | _ | _ | *Bacillus* | | [35] |
| A4 | + | _ | + | _ | K/A | + | + | _ | *Bacillus* | | [35] |
| A5 | + | _ | + | _ | K/K | _ | + | _ | *Bacillus* | | [35] |
| A6 | + | + | _ | + | A/A+G | _ | _ | _ | *Enterobacter* | | [34] |
| A7 | + | + | + | _ | K/A | _ | _ | _ | *Escherichia* | | [36] |
| A8 | _ | + | _ | _ | K/K | _ | _ | _ | *Pseudomonas* | | [37] |
| A9 | _ | + | _ | _ | K/K | + | _ | _ | *Pseudomonas* | | [37] |
| A10 | _ | _ | + | _ | K/K | + | _ | _ | *Pseudomonas* | | [37] |
| S1 | + | _ | + | _ | A/A | _ | _ | + | *Citrobacter* | | [38] |
| S2 | + | + | + | _ | K/A | _ | + | + | *Citrobacter* | | [38] |
| S3 | + | _ | _ | _ | K/K | + | + | _ | *Acinetobacter* | | [39] |
| S4 | _ | + | _ | _ | A/A | _ | + | _ | *Enterococcus* | | [40] |
| S5 | + | + | + | + | A/A | + | _ | _ | *Bacillus* | | [35] |
| S6 | + | _ | + | _ | K/K | + | _ | _ | *Bacillus* | | [35] |
| S7 | + | + | + | + | K/A | _ | _ | _ | *Escherichia* | | [36] |
| S8 | + | _ | + | _ | K/A | + | + | _ | *Bacillus* | | [35] |
| S9 | _ | + | _ | _ | K/A | _ | + | _ | *Enterococcus* | | [40] |
| S10 | + | _ | + | _ | A/A+G | + | + | + | *Proteus* | | [41] |
| TF1 | + | + | _ | _ | A/A | _ | + | _ | *Staphylococcus* | | [42] |
| TF2 | + | + | _ | _ | K/K | _ | + | _ | *Pseudomonas* | | [37] |
| TF3 | + | + | + | _ | K/A | + | + | + | *Citrobacter* | | [38] |
| TF4 | + | _ | + | _ | A/A | _ | _ | _ | *Enterobacter* | | [34] |
| TF5 | + | + | _ | _ | K/K | _ | + | _ | *Bacillus* | | [35] |
| TF6 | + | _ | + | _ | K/A | _ | + | _ | *Enterobacter* | | [34] |
| TF7 | + | + | _ | + | K/A | + | _ | + | *Escherichia* | | [36] |
| TF8 | + | + | _ | _ | K/A | _ | + | _ | *Bacillus* | | [35] |
| TF9 | + | + | _ | + | A/A | + | + | _ | *Escherichia* | | [36] |
| TF10 | + | + | _ | + | A/A | + | + | _ | *Bacillus* | | [35] |
| TF11 | + | + | _ | _ | A/A | ­_ | + | _ | *Staphylococcus* | | [42] |
| TF12 | + | + | + | _ | K/A+G | _ | + | _ | *Enterobacter* | | [34] |
| TF13 | + | + | + | _ | A/A | + | + | _ | *Staphylococcus* | | [42] |
| TF14 | + | _ | + | _ | K/A | + | + | _ | *Staphylococcus* | | [42] |
| TI1 | + | _ | + | + | K/A | + | + | _ | *Bacillus* | | [35] |
| TI2 | + | _ | + | + | A/A | + | _ | + | *Bacillus* | | [35] |
| TI3 | + | + | + | _ | K/A | _ | _ | _ | *Bacillus* | | [35] |
| TI4 | + | + | + | + | A/A+G | ­­_ | ­+ | + | *Proteus* | | [41] |
| TI5 | + | _ | _ | + | A/A | _ | + | _ | *Bacillus* | | [35] |
| TI6 | + | + | _ | _ | K/A | _ | + | _ | *Staphylococcus* | | [42] |
| TI7 | + | _ | + | + | A/A+G | + | + | + | *Proteus* | | [41] |
| TI8 | + | + | _ | _ | K/A | _ | + | _ | *Bacillus* | | [35] |
| TI9 | + | _ | + | _ | A/A | _ | + | + | *Proteus* | | [41] |
| TI10 | + | + | _ | + | K/A | _ | _ | _ | *Escherichia* | | [36] |
| TI11 | + | + | + | _ | K/K | + | + | _ | *Bacillus* | | [35] |
| TE1 | + | + | + | + | A/A | + | + | _ | *Bacillus* | | [35] |
| TE2 | + | + | + | _ | K/A | _ | + | _ | *Bacillus* | | [35] |
| TE3 | + | + | _ | _ | K/A | + | + | _ | *Bacillus* | | [35] |
| TE4 | + | + | _ | _ | K/A | + | _ | + | *Bacillus* | | [35] |
| TE5 | + | + | _ | _ | K/K | _ | ­_ | _ | *Pseudomonas* | | [37] |
| TE6 | + | + | + | _ | A/A | _ | + | _ | *Bacillus* | | [35] |
| TE7 | + | + | + | _ | A/A | _ | + | _ | *Staphylococcus* | | [42] |
| TE8 | + | _ | + | _ | K/K | _ | + | _ | *Pseudomonas* | | [37] |
| TE9 | _ | _ | + | _ | A/A+G | + | + | + | *Proteus* | | [41] |
| TO1 | + | + | + | _ | K/A | + | _ | _ | *Bacillus* | | [35] |
| TO2 | + | _ | + | _ | K/K | _ | _ | _ | *Pseudomonas* | | [37] |
| TO3 | + | + | + | + | K/A | _ | + | _ | *Bacillus* | | [35] |
| TO4 | + | + | _ | _ | K/K | _ | + | _ | *Bacillus* | | [35] |
| TO5 | + | + | + | _ | A/A | + | _ | _ | *Escherichia* | | [36] |
| TO6 | + | + | _ | _ | K/A | _ | + | _ | *Bacillus* | | [35] |
| TO7 | + | + | + | + | A/A | + | + | _ | *Bacillus* | | [35] |
| TO8 | + | + | + | _ | K/A | + | + | _ | *Bacillus* | | [35] |
| TO9 | + | + | _ | + | K/A | _ | + | _ | *Bacillus* | | [35] |
| FI1 | + | _ | + | _ | K/K | _ | _ | _ | *Pseudomonas* | | [37] |
| FI2 | + | + | + | + | K/A | + | + | _ | *Bacillus* | | [35] |
| FI3 | _ | + | _ | _ | A/A | _ | _ | _ | *Enterococcus* | | [40] |
| FI4 | + | + | _ | _ | A/A | + | + | _ | *Bacillus* | | [35] |
| FI5 | + | + | _ | + | A/A | _ | + | _ | *Bacillus* | | [35] |
| FI6 | + | _ | + | _ | K/K | _ | + | _ | *Pseudomonas* | | [37] |
| FO1 | + | _ | + | _ | K/K | _ | + | _ | *Pseudomonas* | | [37] |
| FO2 | + | + | + | + | A/A | + | + | _ | *Bacillus* | | [35] |
| FO3 | + | + | + | _ | K/A | + | + | _ | *Staphylococcus* | | [42] |
| FO4 | + | + | _ | + | K/A | _ | + | _ | *Escherichia* | | [36] |
| FO5 | + | + | _ | _ | K/A | + | _ | _ | *Bacillus* | | [35] |
| FO6 | + | _ | + | _ | K/A | + | + | _ | *Bacillus* | | [35] |
| FO7 | + | + | + | _ | K/A | + | + | _ | *Staphylococcus* | | [42] |
| R1 | + | + | + | + | K/K | _ | + | _ | *Pseudomonas* | | [37] |
| R2 | + | + | + | _ | K/A | _ | + | _ | *Staphylococcus* | | [42] |
| R3 | + | + | _ | _ | K/K | _ | + | _ | *Bacillus* | | [35] |
| R4 | + | + | + | _ | K/A | + | + | _ | *Bacillus* | | [35] |
| R5 | + | + | + | + | K/A | _ | ­_ | _ | *Bacillus* | | [35] |
| R6 | + | + | + | _ | K/A | + | _ | _ | *Staphylococcus* | | [42] |
| R7 | + | _ | _ | _ | A/A | + | + | _ | *Bacillus* | | [35] |
| R8 | + | + | _ | + | K/A | _ | _ | _ | *Bacillus* | | [35] |
| R9 | + | _ | _ | _ | K/K | _ | _ | _ | *Pseudomonas* | | [37] |
| R10 | + | + | + | _ | K/A | + | _ | _ | *Bacillus* | | [35] |
| AB1 | + | + | + | + | K/A | _ | _ | _ | *Escherichia* | | [36] |
| AB2 | + | + | + | + | K/A | _ | _ | _ | *Escherichia* | | [36] |
| AB3 | + | + | + | + | A/A | _ | + | _ | *Bacillus* | | [35] |
| AB4 | + | + | _ | + | K/A | _ | + | _ | *Bacillus* | | [35] |
| AB5 | + | _ | + | _ | K/A | _ | + | + | *Proteus* | | [41] |
| AB6 | + | _ | + | _ | A/K | _ | + | + | *Proteus* | | [41] |
| AB7 | + | + | + | _ | K/A | _ | + | + | *Citrobacter* | | [38] |
| AB8 | + | + | + | + | K/A | + | _ | _ | *Bacillus* | | [35] |
| AB9 | + | + | + | + | K/A | + | _ | _ | *Bacillus* | | [35] |
| AB10 | + | + | _ | _ | A/A | + | _ | _ | *Bacillus* | | [35] |
| AB11 | + | + | _ | _ | K/A | _ | + | _ | *Bacillus* | | [35] |
| AB12 | + | + | + | _ | K/K | _ | + | _ | *Bacillus* | | [35] |
| GU1 | + | + | + | _ | A/A | + | + | _ | *Bacillus* | | [35] |
| GU2 | + | + | + | + | A/A | _ | + | _ | *Escherichia* | | [36] |
| GU3 | + | + | + | _ | K/A | + | + | _ | *Bacillus* | | [35] |
| GU4 | + | + | + | + | A/A | + | + | _ | *Bacillus* | | [35] |
| GU5 | + | + | + | + | A/A | + | _ | _ | *Bacillus* | | [35] |
| GU6 | _ | + | _ | _ | K/A | _ | + | _ | *Enterococcus* | | [40] |
| GU7 | _ | + | _ | _ | A/A | _ | _ | _ | *Enterococcus* | | [40] |

**Note:** A: Animal husbandry; S : Solid waste ; TF: Tannery effluent; TI: Tara gedam inner; TE: Tara gedam edge; TO: Tara gedam outer; FI: Fogera inner; FO: Fogera outer; AB: Abay river mouth; R: Rib river mouth; GU: Gumara river mouth; TSI: Triple Sugar Iron agar; A/A+G: produce both glucose and lactose with Gas; A/K: produce only glucose; K/A: produce only lactose; K/K: not produce both.
